# Dental implant treatment for renal failure patients on dialysis: a clinical guideline

**DOI:** 10.1038/ijos.2017.23

**Published:** 2017-06-23

**Authors:** Quan Yuan, Qiu-Chan Xiong, Megha Gupta, Rosa María López-Pintor, Xiao-Lei Chen, Dutmanee Seriwatanachai, Michael Densmore, Yi Man, Ping Gong

**Affiliations:** 1 State Key Laboratory of Oral Diseases, National Clinical Research Center for Oral Diseases, West China Hospital of Stomatology, Sichuan University, Chengdu, China; 2 Department of Oral Implantology, West China Hospital of Stomatology, Sichuan University, Chengdu, China; 3 Department of Preventive Dental Sciences, Division of Pedodontics, College of Dentistry, Al-Showajra Academic Campus, Jazan University, Gizan, Kingdom of Saudi Arabia; 4 Department of Oral Medicine and Surgery, School of Dentistry, Complutense University, Madrid, Spain; 5 Department of Nephrology, West China Hospital, Sichuan University, Chengdu, China; 6 Department of Oral Biology, Faculty of Dentistry, Mahidol University, Bangkok, Thailand; 7 Department of Oral Medicine, Infection, and Immunity, Harvard School of Dental Medicine, Boston, USA

**Keywords:** chronic kidney disease, dental implant, hemodialysis, osseointegration, renal failure

## Abstract

Chronic kidney disease (CKD) is a worldwide public health problem that is growing in prevalence and is associated with severe complications. During the progression of the disease, a majority of CKD patients suffer oral complications. Dental implants are currently the most reliable and successful treatment for missing teeth. However, due to complications of CKD such as infections, bone lesions, bleeding risks, and altered drug metabolism, dental implant treatment for renal failure patients on dialysis is more challenging. In this review, we have summarized the characteristics of CKD and previous publications regarding dental treatments for renal failure patients. In addition, we discuss our recent research results and clinical experience in order to provide dental implant practitioners with a clinical guideline for dental implant treatment for renal failure patients undergoing hemodialysis.

## Introduction

The number of patients with chronic kidney disease (CKD) is increasing throughout the world.^1, 2^ The global prevalence of CKD is estimated to be 8%–16%,^1^ and the prevalence of CKD varies among countries. The age-standardized global prevalence of CKD in adults is 10.4% for men and 11.8% for women.^3^ For adult US residents, the estimated prevalence of CKD stages 1–4 is 11.6%.^4^ In China, the prevalence of CKD was reported to be 10.8%.^5^ Being older and female are also independently associated with glomerular filtration rate (GFR) and albuminuria in China.^5^ The prevalence of CKD appears to be increasing particularly in older individuals and women.^5, 6^

Some studies have found that 90% of renal disease patients suffer from oral symptoms.^7, 8^ Patients on dialysis may exhibit a variety of oral disorders. The salivary glands, periodontium, teeth, alveolar bone, and oral mucosa can all be affected, leading to oral manifestations, including gingival bleeding, early tooth loss, periodontitis, and xerostomia, among other issues.^9, 10, 11, 12, 13^

Currently, dental implant treatment is the best way to restore missing teeth. However, dental implant treatment in renal failure patients is challenging due to the complications of CKD, such as infections, bone lesions, bleeding risks, and altered drug metabolism. Some researchers have suggested that the use of dental implants and other osseous periodontal surgeries should be very carefully evaluated in renal failure patients.^14, 15^ In fact, we reported that CKD could affect early healing of titanium implants and femoral bone defects in a uremic rodent model.^16, 17^

In this review, we have summarized the characteristics of CKD and discussed the oral manifestations and dental management of these patients. In addition, this research also includes our recent research results and clinical experience to provide dental implant practitioners with a clinical guideline for hemodialysis patients.

## Chronic kidney disease

The decrease in function and structure is reversible in the early stage of CKD. As the disease progresses, the kidneys of patients with CKD are irreversibly altered in function and structure.^18, 19^ The definition of CKD, which was first introduced in 2002, is based on markers of renal damage or on the measurement of a GFR lower than 60 mL·min^−1^ per 1.73 m^2^ for at least 3 months.^18, 20, 21^ CKD was also classified into five stages based on measured or estimated GFR by the Kidney Disease: Improving Global Outcomes (KDIGO) guidelines. These five stages include three early stages ((1) more than 90 mL·min^–1^ per 1.73 m^2^, (2) 60–89 mL·min^−1^ per 1.73 m^2^, and (3) 45–59 mL·min^−1^ per 1.73 m^2^ (stage 3a) and 30–44 mL·min^−1^ per 1.73 m^2^ (stage 3b)) and two advanced stages ((4) 15–29 mL·min^−1^ per 1.73 m^2^ and (5) <15 mL·min^−1^ per 1.73 m^2^).^18, 21^ According to the guidelines published in 2002, the patients in stage 5 CKD are regarded as having kidney failure and need functional replacement by dialysis or transplantation.^18, 19, 20^

### Causes

There are many heterogeneous disease pathways that can cause CKD, although the most frequent causes in all developed and many developing countries are diabetes and hypertension. However, in some countries in Asia and sub-Saharan Africa, glomerulonephritis and unknown causes are more common.^1^ Moreover, diabetic nephropathy is now one of the main causes of end-stage renal disease (ESRD) in some Asian countries, where the proportion exceeds 30%–40%.^22^ In China, chronic glomerulonephritis and diabetes are the main causes of CKD and account for more than 50% of cases of CKD.^1^ Genetic factors can also cause CKD.^23, 24^ Many studies have shown that diabetic patients have a higher risk of oral disorders, and there is a definite relationship between oral disorders and type 2 diabetes.^25, 26, 27, 28, 29^ Currently, compared with patients who have similar GFRs, diabetic patients have an ∼50% higher risk of ESRD.^30^

### Clinical manifestation

Many people with CKD are asymptomatic in its early stages. These patients will not display typical clinical features until later stages of CKD, whereas other patients will exhibit symptoms as a result of kidney damage due to CKD. Owing to the decrease in kidney function, toxins accumulate in the patients’ blood and affect other organs.^31^ The clinical signs and symptoms of renal failure are collectively termed uremia.^11^ Uremia is a state of intoxication that involves multiple extra-renal systems such as the bone, heart, vasculature, and lungs.^32^ Clinical symptoms of renal failure can be readily observed as the whole body and various systems are affected directly and indirectly by the accumulating uremic toxins and their compounds. These include uremic frost, renal osteodystrophy, asterixis, coagulation defects, congestive heart failure, and ammonia taste and breath.^8, 31^ Electrolyte and acid–base disturbances, growth delays in children and hypertension are also observed in these patients.^33^ CKD therapy itself and the associated complications may also cause systemic affects.^31^

### Oral manifestations

CKD patients have many oral manifestations that result from endocrinological imbalances, uremic metabolic disorders, and immunological alterations.^34^ When the stage of CKD advances, both soft and hard tissues can be affected.

#### Oral health status

It has been reported that oral hygiene will decrease in advanced stages of CKD.^35^ On the basis of findings from various researchers, including our own findings, we can say that patients with end-stage kidney disease have poorer oral health status.^9, 12, 36, 37^ Patients on dialysis do brush their teeth once or more daily; however, few use floss, and they tend to make infrequent dental visits.^9, 36^ We identified a study that evaluated the dental status of a group of Chinese patients on hemodialysis and showed that dialysis patients had a great need for dental treatment.^9^ However, our study showed that the number of decayed, missing, or filled teeth was not significantly different between Chinese patients on dialysis and controls.^9^

#### Salivary disorders

Xerostomia is a frequent symptom in hemodialysis patients. Dry mouth is caused by many factors such as reduced salivary flow, minor salivary gland parenchymal fibrosis and atrophy, fluid intake restriction (to maintain a correct fluid volume balance), old age, mouth breathing, and the use of xerostomizing drugs.^8, 10, 11, 38^ Parotitis could appear in CKD due to direct gland involvement, chemical inflammation, side effects of drug therapy, and dehydration, among other causes.^39^ There are relevant studies indicating changes in the components of the saliva of CKD patients. Compared with healthy people, CKD patients have higher concentrations of these factors, including urea, creatinine, sodium, potassium, chloride, and phosphorus, in the saliva.^40, 41, 42, 43, 44^ Increased levels of blood urea nitrogen (BUN) are responsible for high salivary pH and buffering capacity in individuals with CKD.^45^ These patients also receive additional calcium and phosphate supplements, which might account for the high salivary concentrations of these ions.^46^

#### Periodontal condition

Studies have shown that patients on dialysis have poorer periodontal conditions than healthy patients. CKD patients have a higher plaque index and higher dental calculus formation than controls.^47, 48^ Elevated salivary pH, decreased salivary magnesium, and high levels of salivary urea and phosphorus lead to precipitation of calcium–phosphorus and calcium oxalate, and hence dental calculus formation.^49^ However, the conclusions of reports relating to gingival inflammation are controversial.^10, 33^ Some authors reported that gingival inflammation was strongly associated with the formation of dental plaques in CKD patients.^33^ In contrast, other researchers drew the conclusion that there was no obvious relationship.^47, 50^ Severe periodontal destruction may arise in CKD patients.^33, 51^ Compared with the general healthy population, periodontitis and the loss of periodontal bone were significantly more severe in Chinese patients undergoing hemodialysis.^52^ The severe periodontal disease may have a relevant role in the premature tooth loss exhibited by hemodialysis patients.^12^

Patients on hemodialysis have platelet dysfunction due to the dialysis treatment. In addition, anticoagulants such as heparin are used during hemodialysis. Owing to the alteration in coagulation, patients may suffer gingival bleeding.^7, 36^

The most frequently reported periodontal condition is gingival enlargement. Gingival enlargement is a side effect of drugs such as calcium channel blockers, which are frequently used in these patients.^7, 11, 53^ There could be hemodialysis patients who previously received a failed renal transplant, and these patients could suffer from gingival enlargement related to the use of cyclosporine.^10^

Patients may have bad breath and taste because of the presence of urea in the saliva, which is converted to ammonia; this occurs in one-third of hemodialysis patients.^8, 54^

#### Tooth alterations

In children, common oral problems include tooth structure abnormalities,^55^ delayed eruption of permanent teeth, and brown discoloration.^55, 56^ Narrowing or calcification of the tooth pulp chamber can occur in both children^55, 56, 57^ and adults.^57, 58^

It is still controversial, however, whether CKD patients have a higher risk of caries formation. A low caries rate is seen in children with renal disease.^59, 60, 61^ The incidence of dental caries is low in these patients due to the presence of highly buffered and alkaline saliva, which is the result of elevated urea and phosphate concentrations.^50, 56^ Hence, the salivary pH remains above the critical level for demineralization of the dental enamel. Nunn *et al.*^61^ reported a mean decay, missing, filling teeth (DMF) of 0.9 and 0.8 in children suffering from various renal disorders. Nakhjavani and Bayramy^60^ reported a mean DMF score of 2.25 in 5- to 18-year-old children with chronic renal failure in Tehran. There are studies that have shown that the incidence of dental caries appears to be higher in CKD patients; the possible causes are debilitation, hypoplastic enamel, low salivary flow rate, and long-term medication use.^37, 62^ However, other reports have demonstrated the opposite conclusion.^50, 55, 56^ For example, research by Porter *et al.*^63^ research shows that there appears to be no increased risk of cervical caries in hemodialysis patients.

Tooth mobility,^11^ malocclusion, crowding, and severe erosions on the lingual surface of the teeth can also be observed in CKD patients. Finally, patients on hemodialysis have a higher risk of tooth loss than controls.^9, 12^

#### Oral mucosal lesions

The prevalence of oral mucosal lesions is much higher in diabetic patients with end-stage renal failure, which suggests that mucosal lesions can be warning signs for the progression of the disease.^64^ Pallor of the oral mucosa is seen due to reduced erythropoietin (EPO) and the resultant anemia.^65^

In some situations, dialysis and renal transplant patients could suffer lichenoid oral lesions due to drug therapy.^66^ Similar studies report that oral hairy leukoplakia can occur after immunosuppressive drug therapy.^67, 68^ This occurs when BUN levels are above 300 mg·mL^−1^. Kaposi’s sarcoma and non-Hodgkin lymphoma are also observed in immunosuppressed patients.^11, 69^ When the immune system is too weak to fight infection, patients may get candidiasis.^36, 70^ Uremic stomatitis is an uncommon lesion of the oral mucosa associated with advanced renal disease.^71, 72^ Erythematous patch, uremic frost, and ulceration are observed in some patients.^36, 67^

#### Bone-related manifestations

Histological evidence shows that 84% of CKD patients have bone disorders.^73^ Bone metabolism is regulated by several factors including parathormona (PTH), fibroblast growth factor 23 (FGF23), and dihydroxycholecalciferol (1,25(OH)_2_D). Complications from CKD, including hyperphosphatemia, hypocalcemia, hyperparathyroidism, and vitamin D deficiency, may interrupt the balance of these factors, impacting bone structural integrity and resulting in CKD-mineral and bone disorder.^74, 75, 76^

The oral facial disorders related to renal osteodystrophy include bone demineralization, decreased trabeculation, decreased thickness of cortical bone, ground-glass appearance of bone, metastatic soft-tissue calcifications, radiolucent giant cell lesions, radiolucent fibrocystic lesions, lytic areas of bone, jaw fracture, and abnormal bone healing after extraction.^11, 37, 77, 78^ Several cases of expansive jaw lesions have been reported in CKD patients.^78, 79^

## Dental implant treatment

As we previously mentioned, hemodialyzed patients may lose their teeth early due to dental and/or periodontal problems. These patients may go to the dentist asking for implants to replace the missing teeth. Dental implant surgery is complicated for patients with end-stage kidney disease because of the clinical manifestations and side effects of the therapy, including dialysis. Dental implant surgery could also affect the patient. Therefore, dentists need to carefully plan ahead and have a complete plan before conducting the surgery.

### Preoperative period

#### Evaluation of the general condition

Before implant surgery, dentists must evaluate the general condition and oral situation of the patient very carefully. Consultation with the nephrologist is necessary to collect information including the overall degree of CKD, causes, clinical features, risk factors, ongoing therapy, previous and present medical treatments, drug excretion or metabolism, and the best time for implant surgery.^80^ A systemic review should be performed regarding the history of cardiovascular disease and diabetes, immune status or infection, anemia, bone involvement, and abnormal hemostasis.^80^ It is necessary to explain the treatment that we recommend for the patient to the nephrologist in a simple manner.

#### Blood tests

Blood tests should be performed to properly evaluate the patient. The typical preoperative diagnostic testing in patients with CKD includes the measurement of Na^+^, K^+^, Ca^2+^, Mg^2+^, Cl^−^, blood urea, creatinine, and bicarbonate levels. A complete blood count will determine the presence and severity of anemia or thrombocytopenia. The bleeding time should be measured as coagulation should be within the normal limits, and bleeding times of >10–15 min have been associated with high risks of hemorrhage.^81^ If the test results are not normal, it is necessary to refer the patient to a nephrologist to manage the state of the patient. Platelet transfusion should be considered if the platelet count is <50 000/mm^3^.^82^ Platelet dysfunction must be treated with EPO or efficient dialysis. Desmopressin may be used during the perioperative period to temporarily reduce the bleeding time by mobilizing von Willebrand factor.^48^

The following values for important factors should also be requested: PTH; FGF23 (normal level: 33–105 RU·mL^−1^);^83^ and 1,25(OH)_2_D (normal level: 30–100 ng·mL^−1^).^84^ According to the KDIGO guidelines, stage 5 CKD patients should maintain PTH levels between 150 and 300 pg·mL^−1^.^85^ One of the common systemic manifestations of CKD patients is anemia due to the decrease in EPO.^11, 20^ Thus, a complete blood count is necessary to evaluate the patient for possible anemia. Moreover, patients on hemodialysis tend to bleed as a result of platelet dysfunction^86, 87^ and the use of anticoagulants for hemodialysis.^88, 89, 90^ The most common anticoagulant drugs used during dialysis treatment are low-molecular-weight heparin, with a half-life of ~4 h, and heparin, with a half-life of ~1–2 h. Thus, its effect could be minimized if the surgery could be carried out on non-dialysis days.^11, 36^ A commonly accepted practice, in fact, is to perform dental treatment on the day after hemodialysis.^8, 10^ Coagulation tests are needed to ensure that local hemostatic measures are available.^10^

FGF23, which is secreted mostly by mature osteoblasts and osteocytes, has an important role in regulating mineral ion homeostasis.^91, 92^ FGF23 is thought to inhibit mineralization and osteoblast activity.^93, 94^ As renal function declines in patients with CKD, FGF23 levels progressively increase, stimulating phosphaturia and reducing 1,25(OH)_2_D levels, which results in an increase in PTH levels.^95, 96, 97, 98, 99^ The increased FGF23 levels in CKD patients are associated with mortality and vascular calcification, although the direct pathological role of FGF23 is unclear.^100, 101^ Studies carried out by our team demonstrated that FGF23 neutralization improves bone quality and osseointegration of titanium implants in mice with CKD, indicating that FGF23 is a key factor in CKD-related bone diseases. This finding is in agreement with our study and with the findings of other researchers.^102, 103^ 1,25(OH)_2_D, a form of vitamin D, is often deficient in patients with CKD.^104^ 1,25(OH)_2_D insufficiency is defined as 1,25(OH)_2_D levels between 20 and 30 ng·mL^−1^, whereas 1,25(OH)_2_D deficiency is defined as 1,25(OH)_2_D levels below 20 ng·mL^−1^.^84^ In previous studies, a low level of 1,25(OH)_2_D was found to affect bone turnover ratio, mineralization, and metabolism in the bone.^105^ Moreover, it contributes to the appearance of secondary hyperparathyroidism.^106, 107, 108^ Our research group also found that vitamin D supplementation could improve bone–titanium integration in CKD mice.^109^ Other researchers also found that vitamin D might improve various bone properties in diabetic patients.^110^ PTH, 1,25(OH)_2_D, and FGF23 make up a complex, multi-tissue feedback system that can regulate blood phosphate and calcium levels. Therefore, we think that it is necessary to know the levels of these factors to evaluate the patients’ bone metabolism.^111^

#### Evaluation of the residual bone

Evaluation of the residual bone is essential for the success of dental implants in patients with renal failure. In the majority of cases, cone beam computed tomography (CBCT) should be used to develop a three-dimensional view of the residual bone while minimizing radiation exposure unless the diagnostic needs could be met using conventional panoramic X-rays and clinical examination. This examination evaluates the amount of residual bone and provides a better understanding of the intraoral anatomical structures in any given area.^112^ Previously, we used the CBCT scan to assess the residual alveolar bone volume in Chinese CKD patients on hemodialysis.^113^ We found no significant differences in demographics and the extent of tooth loss between patients on hemodialysis and controls. We also found that the residual bone height at the sites of the maxillary premolars and the first molar was significantly lower than that in the control group and that the residual bone width differed depending on the location. Although there are some abnormalities in the mandible and maxilla, the residual alveolar bone is still sufficient for dental implant surgery in hemodialysis patients.^113^

#### Eliminating oral infections

Patients on hemodialysis are at greater risk for infections.^26, 114^ In addition, oral hygiene is also associated with the success of the dental implants. Therefore, it is essential to eliminate plaque biofilm and dental caries before implant treatment. Periodontal treatment is necessary before implant surgery to prevent future peri-implant diseases. In the same way, hemodialysis patients with dental implants should be included in a regular periodontal maintenance program to avoid peri-implant diseases.^6^

#### Planning for the implant surgery

We recommend that the implant surgery should be performed on the first day after hemodialysis, as circulating toxins would be eliminated, intravascular volume is high, and heparin metabolism is at an ideal state. Patients receiving hemodialysis three times a week have an interval of 2 days between sessions. Therefore, in these cases, the implant surgery can also be scheduled for the second day after hemodialysis. The surgery plan should be carefully designed according to the condition of the patient’s residual bone and denture. It is important to have a thorough plan to address the complicated medical situations for patients on dialysis. We also recommend computer-guided flapless surgery for patients with several missing teeth and for patients with complicated situations. The advantage of this approach is avoiding a flap opening, which in turn shortens the operation time, lowers the risk of bleeding, and decreases post-surgical discomfort.

Before implant surgery, the dentist should explain the dental treatment to the patient as well as its possible complications. An informed consent form must be signed by the patient before the surgery.

#### Antibiotic prophylaxis

Patients on hemodialysis are prone to several types of infection due to their immunocompromised status.^26, 114^ A study showed that one-third of renal failure patients suffered from infections.^115^ Infective endocarditis is one of the most common causes of the increased mortality and morbidity in CKD patients.^116^ The American Heart Association (AHA) recommends that prophylactic antibiotics are used before the invasive dental procedures for patients with high risks of infection.^117^ Many drugs are excreted by the kidney; therefore, diminished renal function changes the volume of distribution, metabolism, rate of elimination, and bioavailability of many drugs. Even for drugs metabolized by the liver, renal failure can lead to increased risk of toxicity. Therefore, dentists should avoid excessive accumulation of drugs in patients by lengthening the interval between doses according to the degree of elimination impairment. Nephrotoxic drugs should be avoided entirely.^118^ The choice of antibiotics and dose adjustments should be made based on comments from the patient’s nephrologist before the implant surgery in order to decrease the side effects from CKD. AHA 2007 recommendations suggest that patients should take amoxicillin orally or ampicillin intramuscularly (IM) or intravenously (IV). For patients allergic to amoxicillin, cephalexin and clindamycin can be used. For patients allergic to penicillin and ampicillin or unable to take oral medications, cefazolin and ceftriaxone administered IM or IV can also be considered.^119^ The dose adjustment is associated with the residual kidney function. Aminoglycoside antibiotics and tetracyclines should be avoided in CKD patients due to their nephrotoxicity.^10, 11, 120^ Nitrofurantoin can also produce a toxic metabolite, which can cause peripheral neuritis.^120, 121^ Usually, if the patient is not allergic to penicillin, patients on hemodialysis should take 2 g of amoxicillin orally 1 h before the dental treatment. If the patient is allergic to penicillin, clindamycin is the drug of choice, and 600 mg of clindamycin should be administered orally 1 h before the intervention.^10^

### Perioperative period

#### Monitoring blood pressure

One of the common complications faced by advanced CKD patients is hypertension.^122, 123^ Although patients may take antihypertensive medications, monitoring blood pressure is still necessary. It is suggested that patients undergo dental treatment in the morning. The working environment needs to be quiet, and interruptions must be avoided during the dental procedures.^10, 11^ Sedation may be necessary to reduce anxiety in some cases.^8, 10^

#### Oral antisepsis

Patients are required to rinse with chlorhexidine 0.12%–0.20% mouthwash for 3 min before surgery.

#### Anesthesia and sedation

A safe dosage of local anesthesia is required to perform the surgery. Lidocaine and mepivacaine can safely be used in renal failure patients.^11, 36, 80^ Many patients may have hypertension that can be a cause or complication of CKD. It is necessary to reduce the dose of epinephrine when using local anesthesia due to increasing blood pressure.^36^ Currently, in China, the main anesthetic drug used in dental implant surgery is 4% articaine with epinephrine (1/100 000), which is the same composition as primacaine. For adults, the maximum dose does not exceed 7 mg·kg^−1^.^45^ For anxious patients, we can use topical anesthesia to reduce the pain of the anesthesia injection.^124^ Anxiolytics are indicated in anxious and fearful patients. In these patients, we have to consult the nephrologist to determine the type and dose of anxiolytic agents to use before surgery. Diazepam, midazolam, and other benzodiazepines can safely be used for renal failure patients.^11, 36, 80^ Diazepam is metabolized in the liver, and no dose adjustment is required. The recommended doses for diazepam vary from 0.1 to 0.8 mg per kg of body weight in a single oral dose for conscious sedation.^125^ Midazolam is another drug used in dental sedation that is also metabolized in the liver. The common dosages of midazolam for dental sedation range from 0.5 to 1 mg·kg^−1^ with a maximum of 15 mg.^126^ Nitrous oxide is a colorless, odorless gas that is not metabolized by the human body.^127^ Long-term exposure to nitrous oxide may result in some health problems, including kidney disease.^127^ We did not find any relevant study describing the harmful effects of nitrous oxide administration during conscious sedation in CKD patients (Table 1).

#### Hemostatic measures

A hemostatic plan should be made before the surgery for patients who are prone to excessive bleeding. In addition, a suture must be used when the gingival margins do not oppose well. Comments from the nephrologist should be used to make a plan for surgical hemostatic measures. Common local hemostatic measures, including mechanical compression, packing, suturing, and topical thrombin, should be used as often as possible for patients with a risk of bleeding.^36, 88, 128^ Moreover, conjugated estrogen can reverse any platelet dysfunction and may be used for long-term hemostasis for up to 2 weeks.^129^ Desmopressin can be utilized in cases of severe bleeding for patients with renal failure.^80^ Furthermore, tranexamic acid has been shown to reduce bleeding during and after surgery.^130, 131^ Lockhart *et al.*^132^ suggest the use of electrocautery to control hemorrhage during invasive dental procedures.

#### Minimally invasive procedures for implant surgery

As has been shown, the use of minimally invasive procedures can reduce patient pain and shorten recovery time. Moreover, minimally invasive procedures can also decrease the risk of bleeding and infection. Using a template for placement of the implant may be less invasive than traditional methods.

### Post-operative period

#### Regular advice for patients

The dentist should give the patient thorough post-operative instructions. Smoking, mouth rinsing, and strenuous activities must be avoided for 24 h. A soft-food diet is recommended for the first 24 h. Following the dentist’s advice, the patient must take medications as directed. Antibacterial mouthwash (chlorhexidine 0.12% twice daily) is required for at least a week. The dentist should give the patient emergency contact information in case emergencies occur after the implant surgery.^133^

#### Antibiotic treatment

It is not clear whether post-operative infections and implant failure can be reduced with the use of antibiotics.^46^ Moreover, no consensus exists about the appropriate dosage regimen in implant dentistry. As hemodialyzed patients are compromised patients and are subjected to numerous blood exchanges, leading to an increased risk of infections, antibiotic treatment after implant surgery may be recommended. The type and dose of antibiotic should be determined by the nephrologist. If amoxicillin is used, it is necessary to adjust the dose (Table 1).

#### Analgesics and anti-inflammatory drugs

After implant surgery, especially for the first few days, it is often necessary to use analgesics to control pain. Paracetamol is the most frequently recommended analgesic for pain in dialyzed patients (see doses in Table 1).^134^

The use of nonsteroidal anti-inflammatory drugs (NSAIDs) is still controversial in patients with CKD and should be assessed by the nephrologist when excess inflammation is expected after surgery. Some clinicians recommend dose adjustments when these drugs are used for renal failure patients because they inhibit prostaglandins and generate a hypertensive effect.^10, 11^ Short-term use of NSAIDs is generally safe in early-stage CKD patients without heart failure, diabetes, or hypertension.^135^ However, others have suggested avoiding the use of NSAIDs for CKD patients whenever possible.^136^

Aspirin should be avoided in uremic patients due to its antiplatelet activity.^10, 11^ Meperidine, dextropropoxyphene, morphine, tramadol, and codeine can cause central nervous system hypofunction and have respiratory effects due to accumulation in patients with CKD.^13, 120^

### Restoration

#### Timing for restoration

The success of the dental implant depends on osseointegration. In a previous study, we explored the effects of CKD on the osseointegration of titanium implants in a uremic mouse model and compared the results with those in control mice.^17^ We found that there was a significant difference in biomechanical resistance at the early healing stage (2 weeks) between uremic mice and control mice. However, all implants in the CKD mice reached osseointegration successfully after 4 weeks.^17^ Currently, there is no relevant clinical research that indicates that CKD can affect implant healing. We suggest that dentists should extend the healing time or use a temporary crown with lower occlusal force before the final restoration.

#### Evaluation of osseointegration

Implant stability is a prerequisite for a successful clinical outcome. There are several methods to evaluate the stability of a dental implant in the dental clinic. One of these methods is the resonance frequency analysis-reliability system developed by Osstell (Columbia, MD, USA) that is a reliable instrument for estimating the stability of dental implants.^137^ Oral radiography is also a direct and reliable way to evaluate osseointegration.^138^

It is very important to quantify implant stability at various time points and to project a long-term prognosis based on measured implant stability in dialyzed patients due to the possible alteration in bone structural integrity.

#### Retention of the restorations

As we previously observed, patients on dialysis have poor oral health and are more likely to get oral infections and periodontitis.^9, 10, 11^ Gingival enlargement due to calcium channel blockers is one of the most common oral symptoms in patients with renal failure.^11, 132^ Gingival enlargement could appear around dental implants. Many dialysis patients are on the waiting list for a kidney transplant. We have to consider that kidney transplants are treated with immunosuppressants. Some immunosuppressants such as cyclosporine A and, according to some authors, tacrolimus are also associated with gingival enlargement.^53, 139^ Proper periodontal maintenance is necessary to avoid the appearance of gingival enlargement. Despite proper periodontal control, gingival enlargement may appear around the implants. It is necessary to consult with the nephrologist in order to exchange a calcium channel blocker for another antihypertensive medication. In the case of gingival enlargement associated with cyclosporin A, it would be necessary to consider exchanging cyclosporin A for tacrolimus because tacrolimus is less frequently associated with this side effect.

Considering these possible risk factors, we highly recommend screw retention for ease of maintenance. Screw retention is designed so that it is easy and safe to remove the crown, which is convenient for maintenance of the implant.

### Maintenance

#### Risk factors of dental implant failure

Smoking is a significant risk factor that can increase early dental implant failure.^140^ Therefore, smoking should be avoided by CKD patients with dental implants. In addition to smoking, a history of periodontitis, poor oral hygiene, systemic disease, soft-tissue defects, and a history of dental implant failure are also risk factors that may result in peri-implantitis.^141^ The oral cavity of CKD patients on dialysis should be examined carefully and frequently for prompt diagnosis and treatment of possible dental implant diseases and other oral problems.

It is important to remember that vitamin D deficiency may also have a role in osseointegration failure.^142^ Thus, appropriate vitamin D supplementation may help with osseointegration.^109^

#### The prevention and therapy of peri-implant mucositis and peri-implantitis

Peri-implantitis is defined as the inflammation and destruction of soft and hard tissues surrounding dental implants.^141, 143^ Peri-implant mucositis is defined as a reversible inflammatory process of the peri-implant soft tissue.^141, 143^ The prevalence of peri-implantitis was found to vary from 5 to 63.4% across several studies.^141^

Microbial biofilm is commonly considered as the key factor in the development of peri-implantitis.^144^ Therefore, to prevent peri-implant diseases in hemodialysis patients, it is necessary to include the patients in a regular periodontal maintenance program and to recommend proper oral hygiene on a regular basis.^6^ In addition, it has been observed that systemic diseases such as diabetes, cardiovascular diseases, and/or osteoporosis can have important roles in periodontal disease. Therefore, the proper control of these systemic diseases can help prevent periodontal problems and peri-implant diseases.^145, 146^ When peri-implantitis occurs, patients can undergo both nonsurgical and surgical treatments that are very similar to the treatments available for periodontitis.^144^

The main therapy for mucositis and peri-implantitis is the nonsurgical approach, which includes manual treatment, drug therapy, laser therapy, and photodynamic therapy. A significant finding is that nonsurgical periodontal therapy was sufficient to improve the periodontal condition of ESRD patients, and this therapy has beneficial systemic effects in these patients.^147^

When nonsurgical treatment fails, other available therapies for peri-implantitis include surgical approaches such as resective therapy and regenerative approaches. However, a surgical approach should not be used exclusively, but rather, in combination with nonsurgical methods. The recommendations for surgical treatment are the same as those for implant surgery that were detailed above.

## Conclusion

In conclusion, the number of patients on dialysis is progressively increasing. These patients may require implant treatment to restore their missing teeth and improve their quality of life. The systemic condition of these patients and the dialysis treatment can complicate the implant treatment. Therefore, the treatment must be carefully planned using the simplest possible treatment to avoid possible complications and modifying the dosage schedule of the drugs used as necessary for each patient. The oral health status of dialysis patients should be reviewed carefully and frequently for prompt diagnosis and treatment of possible peri-implant diseases and other oral problems. Further work is needed to evaluate the success of implant treatment in these patients. The recommendations for conducting implant surgery discussed in this review will be helpful to clinicians for planning and conducting implant procedures in CKD patients.

## Figures and Tables

**Table 1:** Dose adjustment for patients on dialysis

Drug species	Common dose	Adjustment method
Antibiotics		
Amoxicillin	250–500 mg every 8 h	Prolongation of the dosing interval every 24 h
Doxycycline	No adjustment needed	—
Erythromycin	No adjustment needed	—
Tetracycline	250–500 mg two to four times daily	Prolongation of the dosing interval every 24 h
Clindamycin	No adjustment needed	—
Ampicillin	1–2 g ampicillin and 0.5–1 g sulbactam every 6–8 h	Prolongation of the dosing interval every 12–24 h
Aciclovir	200–800 mg every 4–12 h	Prolongation of the dosing interval 200 mg every 12 h
Ketoconazole	No adjustment needed	—
		
Anesthetics		
Lidocaine	No adjustment needed	—
Mepivacaine	No adjustment needed	—
Articaine	No adjustment needed	—
		
Sedation		
Codeine	Not recommended	—
Alprazolam	Not recommended	—
Diazepam	No adjustment needed	—
Midazolam	No adjustment needed	—
		
Analgesics		
Aspirin	Avoid	—
Ibuprofen	Avoid	—
Diclofenac	Avoid	—
Paracetamol	300–600 mg every 4 h	Prolongation of the dosing interval every 8–12 h
